# A Comprehensive Analysis of Histopathologic Examination Results of Tonsillectomy Specimens

**DOI:** 10.7759/cureus.6655

**Published:** 2020-01-14

**Authors:** Serkan Kayabasi, Omer Hizli, Serkan Cayir, Melike Ordu

**Affiliations:** 1 Otolaryngology, Faculty of Medicine, Aksaray University, Aksaray, TUR; 2 Otolaryngology, Prof. Dr. A. Ilhan Ozdemir Education and Research Hospital, Giresun University, Giresun, TUR; 3 Otolaryngology, Aksaray Education and Research Hospital, Aksaray University, Aksaray, TUR; 4 Pathology, Faculty of Medicine, Aksaray University, Aksaray, TUR

**Keywords:** tonsillectomy, histopathology, malignancy, asymmetry, hypertrophy

## Abstract

Objective

This study aims to review the histopathologic results of tonsillectomy specimens, determine the rates of the tonsillectomy indications, and investigate the characteristics of asymmetric hypertrophy.

Materials and Methods

Medical records of 484 patients who underwent tonsillectomy were reviewed retrospectively. Descriptive data of adult and pediatric patients were presented as percentage. Comparisons between asymmetric and symmetric hypertrophy groups were performed to determine the features of asymmetric hypertrophy.

Results

The mean age of 484 patients who underwent tonsillectomy was 13 years (range: 3-69 years). While 372 (76.85%) patients were operated for infection, 100 (20.66%) were operated for tonsillar hypertrophy, 1 (0.21%) for a suspicion of malignancy, and 11 (2.27%) for other various reasons. Asymmetric hypertrophy was seen in 25 (5.16%) patients, whereas symmetric hypertrophy was seen in 75 (15.49%) patients. Malignancy was detected in three (0.61%) adult patients with asymmetric hypertrophy. Tonsillar tuberculosis was observed in one foreign patient with asymmetric hypertrophy. The presence of malignancy was higher in the asymmetric hypertrophy group (three patients [12%]) compared with the symmetric hypertrophy group (none) (p=0.002; X^2^=9.27). Median maximum specimen diameter was 3 cm (range: 1.15-5.5 cm) in the asymmetric hypertrophy group and 2.4 cm (range: 1.25-4.8 cm) in the symmetric hypertrophy group (p=0.08). The Friedman grade was significantly (p<0.001), positively, and strongly (r=0.885) correlated with the maximum specimen diameter.

Conclusion

Routine histopathologic examination of the tonsillectomy specimens might not be necessary for all patients, but it is recommended for the patients with a real asymmetry.

## Introduction

Tonsillectomy is one of the most often performed operations in daily otolaryngology practice [1]. Tonsillectomy indications can be categorized as relative and definite. Recurrent tonsillitis, obstructive sleep apnea, peritonsillar abscess, halitosis, and suspicion of malignancy are the most common tonsillectomy indications. The most common indication in adults might be chronic recurrent tonsillitis; however, tonsillectomy has been gradually further performed as a part of the surgical treatment of obstructive sleep apnea in recent years [2]. There is no clear consensus on whether the histopathologic examination of clinically good-looking tonsil specimens is required [3]. Tonsillar malignant tumors might be clinically suspected of, but biopsy is required for certain histopathologic diagnoses. Therefore, tonsillectomy is usually performed for biopsy purposes in patients with a suspicion of malignancy. In the studies with large series, the risk of occult malignancy was reported to be quite lower in children [4]. The most important malignancy observed in children is usually tonsillar non-Hodgkin’s lymphoma [5]. In adult patients, the incidence of malignancy after tonsillectomy varies from 2% to 10% [6]. The most common tonsillar malignancy is squamous cell carcinoma, which constitutes the majority of cases [7]. While squamous cell carcinoma occurs with an ulceration over the tonsil, basal cell carcinoma usually occurs with a normal mucosal and tonsillar asymmetry. Diagnosis of malignancy is very difficult in patients with normal tonsillar mucosa and without cervical adenopathy and/or additional physical examination findings [8].

In this study, we evaluated the histopathologic examination results of the patients who underwent tonsillectomy and determined the rates of the indications for tonsillectomy. The aim of the study was to determine the prevalence of various significant pathologies in patients with clinically asymmetric tonsils and to discuss the necessity of diagnostic tonsillectomy in patients with asymmetric tonsils.

## Materials and methods

Study Design

This retrospective study was conducted in line with the dictates of the World Medical Association Declaration of Helsinki and was approved by the local ethical committee (IRB Number: E-18-2420). The study included 484 patients who underwent tonsillectomy due to various indications (968 specimens) between April 2012 and January 2019. The ages, genders, tonsillectomy indications, histopathologic examination results, and clinical findings of the patients were noted by reviewing the medical records. According to the preoperative physical examination of all patients, the size of the tonsil was graded based on the grading system described by Friedman et al. [9]. According to this system, 0 (inside the tonsillar plica) was considered as atrophy, 1+ (at the same level as the tonsillar plica) was considered as normal, and 2+, 3+, and 4+ (tonsils that close the oropharyngeal passage more than 25%, 50%, and 75%, respectively) were considered as hypertrophy. Those with a difference of grade over 1+ between the right and left sides were considered as asymmetric tonsillar hypertrophy (ATH). The surgical tonsillectomy method was cold dissection or thermal welding. Macroscopic and light microscopic histopathologic examination of the left and right tonsils was performed separately. The maximum diameter values of specimens were obtained from histopathologic examination result reports.

Tonsillectomy indications were categorized as chronic and/or recurrent infection, upper airway obstruction, suspicion of malignancy, and other reasons [1]. Patients with seven or more attacks of tonsillitis in the past year, those with five or more attacks of tonsillitis in the past two consecutive years, and those with three or more attacks of tonsillitis in the past three consecutive years were included in the first group as recurrent tonsillitis. The patients with snoring, open mouth sleeping, cessation of breathing during sleep in the non-infectious period, three or three attacks of tonsillitis in a year, and asymmetric or symmetric hypertrophy were included in the second group. The patients who were suspected of malignancy were included in the third group. Rare cases such as halitosis, tonsillolithiasis, tonsillar cyst, and tonsil bleeding were considered as the other reasons.

We constituted two groups by reference to the presence of asymmetric hypertrophy: ATH group and symmetric tonsillar hypertrophy (STH) group. We compared the presence of malignancy, actinomycosis, reactive lymphoid hyperplasia, and chronic inflammation between the groups. Additionally, we compared the mean ages, gender distribution, and median maximum specimen diameters of the groups. Also, to investigate whether preoperative Friedman grading was accurate, we performed a correlation test between the Friedman grade and maximum specimen diameter.

Statistical Analysis

We presented the results as median (min-max) and percentage (%). We confirmed the abnormal distribution of data using the Kolmogorov-Smirnov normality test. To investigate the presence of malignancy, actinomycosis, reactive lymphoid hyperplasia, and chronic inflammation, we used the chi-square test. To compare the maximum specimen diameters of the groups, we used the Mann-Whitney U test. To investigate the correlation between the Friedman grade and maximum specimen diameter, we used Spearman’s correlation test because Friedman grade data were ordinal. To compare the difference of maximum diameters of the right and left tonsil specimens (addressing asymmetry), we used the Mann-Whitney U test. We performed all statistical analyses using SPSS 16.0 software for Windows (SPSS Inc., Chicago, IL, USA). A p-value of less than 0.05 was considered statistically significant.

## Results

The mean age of 484 patients was 13 years (range: 3-69 years), Of whom 282 (58.6%) were males and 202 (41.7%) were females. Of the patients, 411 (84.91%) were under the age of 18 years (pediatric group), and 73 (15.08%) were over the age of 18 years (adult group). The mean ages of children and adults were 7.1 years (range: 3-17 years) and 29.3 years (range: 18-69 years), respectively.

While 372 (76.85%) patients underwent tonsillectomy for chronic/ recurrent infection, 100 (20.66%) were operated for tonsillar hypertrophy, 1 (0.21%) for a suspicion of malignancy, and 11 (2.27%) for other reasons. The distribution of tonsillectomy indications in the pediatric and adult patients are shown in Table 1.

**Table 1:** The distribution of tonsillectomy indications in the pediatric and adult patients

Indications	Pediatric patients		Adult patients	
	n	%	n	%
Infections	312	75.91%	60	82.2%
Obstruction	95	23.12%	5	6.85%
Suspicion of malignancy	0	0%	1	1.37%
Other reasons	4	0.97%	7	9.58%
Total	411	100	73	100

Among the patients operated for tonsillar hypertrophy, ATH was detected in 25 (5.16%) patients, whereas STH was detected in 75 (15.49%) patients. Adult tonsillar squamous cell carcinoma was detected in one (0.21%) patient operated for a suspicion of malignancy. With respect to the other indications, two patients (0.41%) had tonsil bleeding, three (0.61%) had tonsillolithiasis with halitosis, three (0.61%) had tonsillar papilloma, and three (0.61%) had tonsil cyst.

**Figure 1:**
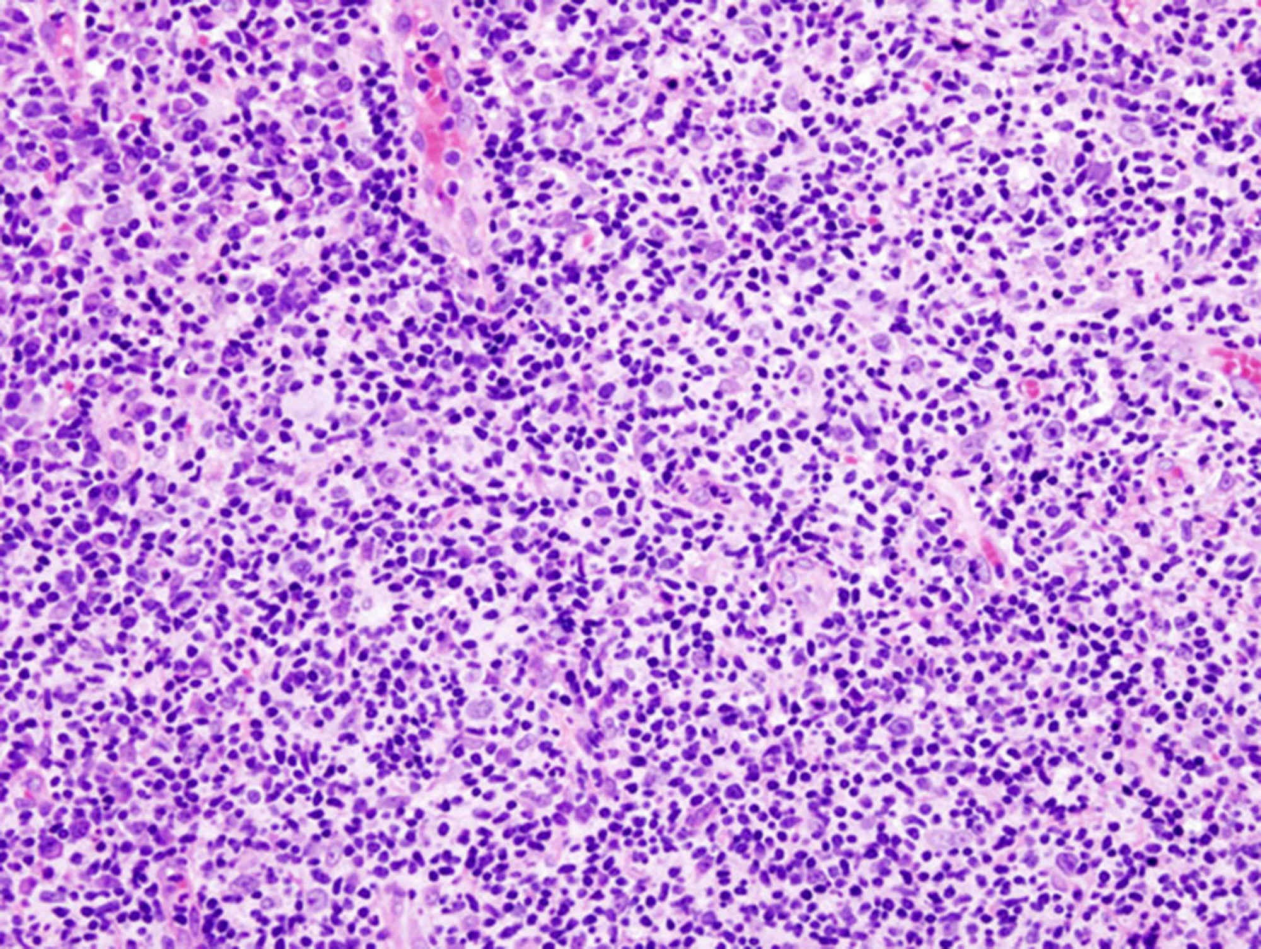
Light photomicrograph of histopathologic section from a patient with diffuse large B-cell non-Hodgkin lymphoma (hematoxylin and eosin stain, 100x magnification)

Of 484 (968 specimens) patients who underwent tonsillectomy, 219 (45.24%) had reactive lymphoid hyperplasia, 137 (28.30%) had reactive lymphoid hyperplasia with chronic inflammation, and 97 (20.04%) had chronic inflammation in their histopathologic examination result reports. Malignancy was detected only in the specimens of three (0.30%) patients. All these patients were in the adult patient group. Malignancy was not evident in the pediatric patient group. Two patients with malignancy were diagnosed as having diffuse large B-cell non-Hodgkin lymphoma (Figure 1).

**Figure 2:**
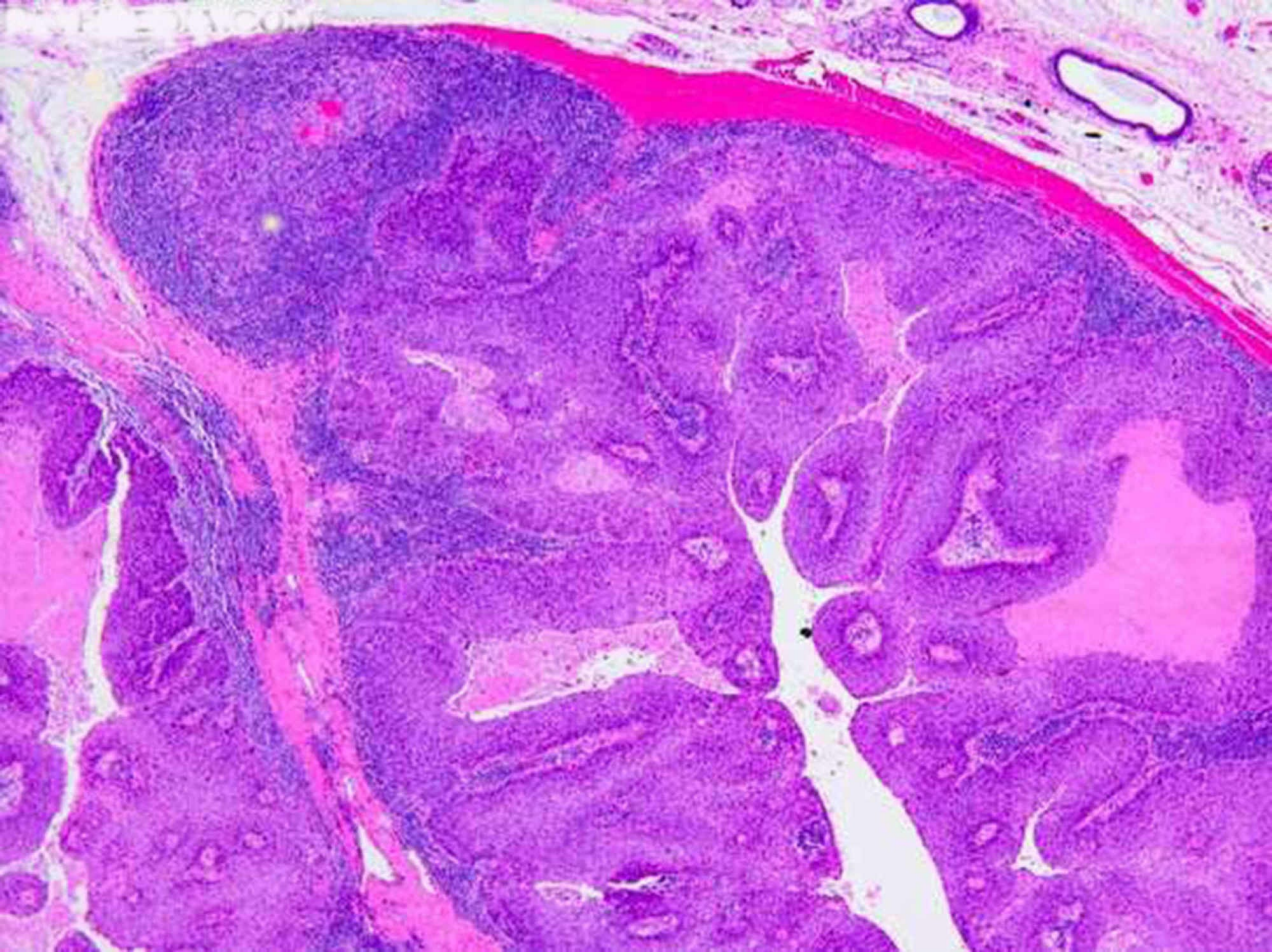
Light photomicrograph of histopathologic section from the patient with squamous cell carcinoma (hematoxylin and eosin stain, 10x magnification)

While unilateral tonsillar hypertrophy (asymmetric hypertrophy) was detected in these patients with lymphoma, normal tonsillar mucosa without any ulceration was seen in physical examination. The first patient was a 54-year-old woman with recurrent attacks of tonsillitis and left tonsillar hypertrophy. The other patient was a 64-year-old woman with right tonsillar hypertrophy. Both patients underwent tonsillectomy for recurrent tonsillitis, not for any suspicion of malignancy. Thus, two (0.41%) patients in our study group had an occult malignancy (without a preoperative suspicion). Additionally, occult malignancy was not seen in pediatric patients, and the incidence of occult malignancy was 2.7% in adult patients. In the physical examination of both patients, preoperative palpable lymphadenopathy findings were remarkable. In addition to the routine histopathologic examination, immunohistochemical evaluation was performed in both patients. The immunohistochemical staining revealed positive keratin, negative CD 20, and negative CD3 staining features in both patients. Out of 484 patients, only one (0.21%) patient underwent tonsillectomy for a suspicion of malignancy. In the physical examination of this patient, ulceration and ATH were seen. Histopathologic examination result of this 69-year-old male patient was reported as squamous cell carcinoma (Figure 2).

**Figure 3:**
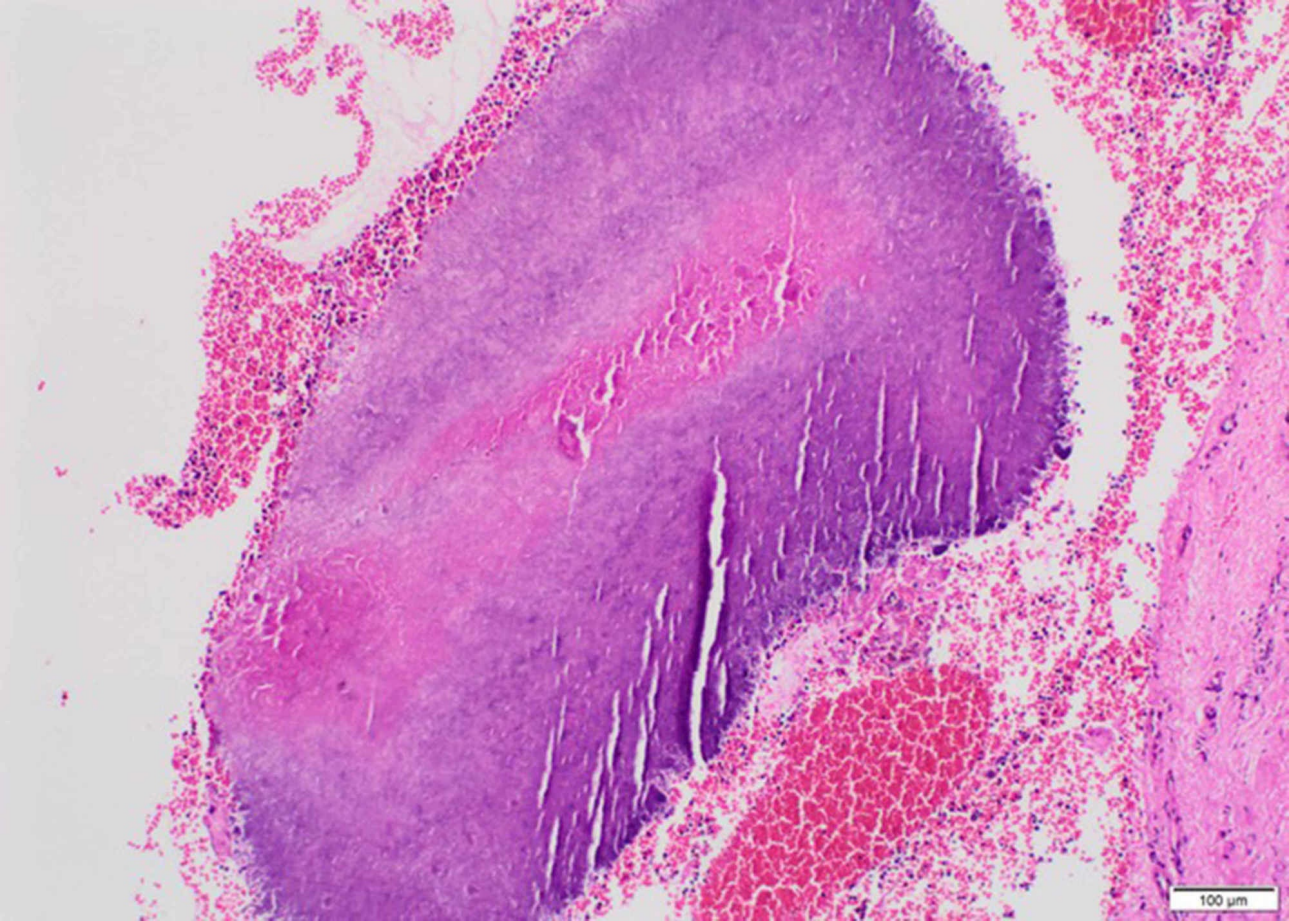
Light photomicrograph of histopathologic section from the patient with tonsillar actinomycosis (hematoxylin and eosin stain, x10 magnification)

According to the analysis of histopathologic examination results, 22 patients had actinomycosis (14 pediatric patients and 8 adult patients) (Figure 3), 3 had squamous papilloma (2 pediatric patients and 1 adult patient), and 3 had epidermal inclusion cyst (2 pediatric patients and 1 adult patient). One adult patient with ATH had tonsillar tuberculosis (Table 2).

**Table 2:** Histopathologic examination results of our study population

Histopathologic diagnosis	Pediatric patients		Adult patients		Total	
	n	%	n	%	n	%
Chronic inflammation	70	17.03	27	36.98	97	20.04
Reactive lymphoid hyperplasia	205	49.87	14	19.17	219	45.24
Chronic inflammation + reactive lymphoid hyperplasia	118	28.71	19	26.02	137	28.30
Malignancies	-	-	3	2.19	3	0.61
Actinomycosis	14	3.40	8	10.95	22	4.54
Tuberculosis	-	-	1	1.36	1	0.20
Squamous papilloma	2	0.48	1	1.36	3	0.61
Epidermal inclusion cyst	2	0.48	1	1.36	3	0.61
Total	411	84.91	73	15.08	484	100

Out of 484 patients who underwent tonsillectomy, 100 (20.6%) had tonsillar hypertrophy. The ATH group consisted of 25 patients (13 males and 12 females; mean age: 16 years [range: 3-69 years]), and the STH group consisted of 75 patients (41 males and 34 females; mean age: 11 years [range: 3-44 years]). The groups were age and gender-matched (p=0.82 and p=0.81, respectively). While no (0%) malignancy was reported in histopathologic examination results of the patients from the STH group, three (12%) malignancies were reported in the ATH group (X2=9.27; p=0.02). One (4%) patient in the ATH group and six (8%) patients in the STH group had actinomycosis, thus not significantly different (X2=0.46; p=0.49). Reactive lymphoid hyperplasia was present in 17 (68%) patients in the ATH group and 61 (81.3%) patients in the STH group, also not significantly different (X2=1.94; p=0.16). Chronic inflammation was present in 8 (32%) patients in the ATH group and 34 (45.3%) patients in the STH group, again not significantly different (X2=1.36; p=0.24) (Table 3). Thus, ATH was associated with tonsillar malignancies but not with actinomycosis, reactive lymphoid hyperplasia, and chronic inflammation.

**Table 3:** Comparison of clinicopathological findings of the groups ATH, asymmetric tonsillar hypertrophy; STH, symmetric tonsillar hypertrophy

Clinicopathological findings	ATH group, n=25	STH group, n=75	p-Value	X^2 ^value
Age, years	16 (3-69)	11 (3-44)	0.82	
Gender (male/female)	13/12	41/34	0.81	
Median maximum specimen diameter (cm)	3 (1.15-5.5)	2.4 (1.25-4.8)	0.008	
Median difference of maximum specimen diameter between the right and left tonsils (cm)	1.20 (0.5-3.4)	0.15 (0-1.45)	<0.001	
Malignancy	3 (12%)	0 (0%)	0.02	9.27
Chronic inflammation	8 (32%)	34 (45.3%)	0.24	1.36
Reactive lymphoid hyperplasia	17 (68%)	61 (81.3%)	0.16	1.94
Actinomycosis	1 (4%)	6 (8%)	0.49	0.46

The Friedman grade was significantly (p<0.001), positively, and strongly (r=0.885) correlated with the maximum specimen diameter of tonsils. The median maximum specimen diameter was 3 cm (range: 1.15-5.5 cm) in the ATH group and 2.4 cm (range: 1.25-4.8 cm) in the STH group. Patients with ATH had significantly greater maximum specimen diameter (p=0.008). The median difference of maximum diameters between the right and left tonsils was 1.20 cm (range: 0.5-3.4 cm) in the ATH group and 0.15 cm (range: 0-1.45 cm) in the STH group, thus significantly greater in the ATH group (p<0.001) as expected.

## Discussion

Tonsillectomy (with or without adenoidectomy) is one of the most common operations performed in children [10]. Tonsillectomy has been previously performed to avoid the complications of group A beta-hemolytic streptococcal infection; however, it is now performed mainly for chronic and/or recurrent infection and obstructive reasons [11]. In this study, while 372 (76.85%) patients underwent tonsillectomy due to the diagnosis of chronic and/or recurrent infection, 100 (20.66%) underwent tonsillectomy for upper airway obstruction due to tonsillar hypertrophy.

Obstructive causes were reported to increase in recent years among tonsillectomy indications, particularly in pediatric patients. According to the report by Parker and Walner, obstruction was the primary tonsillectomy indication (91.8%) in the 0-3 age group, of which only 7.5% underwent tonsillectomy for chronic recurrent infection. [12]. In contrast to the prior literature, 75.91% of pediatric patients underwent tonsillectomy due to the diagnosis of chronic and/or recurrent infection, whereas 23.12% underwent tonsillectomy due to the diagnosis of upper airway obstruction in our study. Similarly, the most common tonsillectomy indication was infectious causes in our adult patient group (60 [82.2%] patients).

According to the prior literature, the most common tonsillar malignancy was squamous cell carcinoma in adults. Lymphomas were more frequently seen in children. The incidence of occult malignancy was reported as 0-1% in the literature [13]. In our study, malignancy was observed in one (0.21%) patient who underwent tonsillectomy due to a suspicion of malignancy. This patient was a 69-year-old man with ATH, and his histopathologic examination result was reported as squamous cell carcinoma. The incidence of tonsillar malignancy was higher in adults compared with children, which might be due to smoking, alcohol consumption, and other environmental factors. In our study group, occult malignancy was seen in two (0.41%) patients, consistent with the prior literature [13].

In our study, an epidermal inclusion cyst was detected in one adult patient and squamous papilloma was detected in another adult patient. Actinomycosis with chronic inflammation was reported in eight adult patients. Tonsillar tuberculosis was detected in a foreign adult with ATH. Tonsillar tuberculosis is an extrapulmonary type of tuberculosis that can frequently mimic tonsillar malignancy and is rarely observed (10-15%) [14]. Its secondary form is more common than its primary form. Contact with the infected sputum or saliva is the main source of the disease. Clinical and recurrent tonsillitis is the main clinical presentation with large asymmetric tonsils and sore throat. Since it is very difficult to distinguish it from malignancy clinically, the histopathologic examination of the tissue is required for certain diagnoses. Tonsillar tuberculosis is also not a common entity in Turkey; thus, our results suggest that migrations and exchanges may affect the infectious disease profile in a country.

Malignancy was not observed in our pediatric patient group. In the previous reports, malignancy rates ranged from 0% to 0.17% in the pediatric population [15]. Although tonsillar malignancy rates in pediatric patients are lower, a detailed histopathologic examination should be performed in patients with preoperative risk factors such as necrotic tonsil, lymphadenopathy on the neck, weight loss, night sweating, and tonsillar asymmetry. In our study, histopathologic examination was reported as epidermal inclusion cyst in two pediatric patients and as squamous papilloma in two pediatric patients. These patients had preoperative well-defined benign lesions, and histopathologic findings were consistent with clinical findings. The presence of actinomycosis in tonsil specimens was reported as 1.3-57% [16]. In our study, actinomycosis with chronic inflammation was observed in 14 pediatric patients. Many previous studies claimed that actinomycosis might play a role in the etiology of chronic tonsillitis and adenoid vegetation, although it was not definitely demonstrated [17].

Although histopathologic examination is usually performed after tonsillectomy in general practice, the necessity of histopathologic examination is a matter of debate. In addition, it is still unclear that in which cases histopathologic examination is required. Felippe et al. reviewed 2013 adult and pediatric patients who underwent adeno-tonsillectomy and reported malignancy in 4 (0.19%) patients [18]. In the report by Younis et al., malignancy was not detected in 2099 pediatric patients and was detected in 40 (11.79%) of 339 adult patients. The most common malignancy type was squamous cell carcinoma in this report [19]. In addition, they suggested that gross examination of specimens was sufficient and they required histopathologic examination in suspected cases [19]. In our study, malignancy was detected in two patients who underwent tonsillectomy for obstruction. These patients were in the adult group and diagnosed as having diffuse large B-cell non-Hodgkin lymphoma. Tonsillar asymmetry and lymphadenopathy on the neck were present in both patients, and normal mucosa without ulceration was seen in their physical examinations. These two patients with lymphoma could be considered as occult malignancy due to the absence of preoperative suspicion of malignancy. Based on our results, we can suggest that histopathologic examination is needed for the specimens of adult patients with ATH and any additional physical examination finding, such as lymphadenopathy of the neck, due to occult malignancy risk.

ATH is not a rare condition. Even though ATH is usually benign, tonsillar tumors should be taken into consideration at differential diagnosis. In our study, no malignancy was evident in patients with STH. Chronic inflammation, reactive lymphoid hyperplasia, and actinomycosis rates were higher in the STH group compared with the ATH group but did not significantly differ. This difference might have been significant if the ATH group consisted of a larger number of patients. Therefore, we found that ATH was associated with tonsillar malignancies in this study, but the evidence of association of STH with actinomycosis, reactive lymphoid hyperplasia, and chronic inflammation was not clear. Spinou et al. reported the malignancy rate of the patients with ATH as high as 23.4%, as 23 malignancies were present in 98 adults with ATH in their study. Accordingly, they found a clinical suspicion of malignancy in each of these cases, such as ulceration, male gender, lymphadenopathy, age over 45 years, smoking, and weight loss [20].

In our study, we found a significant and strong correlation between the preoperative grade and maximum specimen diameter of excised tonsils. However, Cable et al reported that clinically assessed size was not correlated with the volume pathologically measured after tonsillectomy in children [21]. Berkowitz and Mahadevan found that only 52% of histopathologic examination results revealed a tonsillar size asymmetry in patients considered to have a preoperative large ATH [22]. According to Spinou et al., 17% of the patients with clinically asymmetric tonsils had a smaller tonsil size and that the sizes of the right and left tonsils were equal in 40% of the patients [23]. Cinar did not find a difference in the tonsil size of 39.62% of the asymmetric group and concluded that apparent asymmetry might be due to the difference in the tonsillar fossa depth [8]. In contrast to these studies, Syms et al. reported that clinically assessed size was consistent with pathologically measured volume in 60.5% of the patients with ATH [24]. In our study, patients with ATH had significantly greater maximum specimen diameter compared with those with STH. Furthermore, the Friedman grade was significantly, positively, and strongly correlated with the maximum specimen diameter of the tonsils. This result shows that we performed an accurate preoperative Friedman grade evaluation or that the Friedman grading system [9] might be considered as an accurate grading system.

## Conclusions

In conclusion, the routine histopathologic examination of the tonsillectomy specimens might not be necessary for all patients, but it is recommended for patients with a real asymmetry. Physicians should bear in mind the possibility of asymmetry due to variability in the tonsillar fossa. Cooperation of otolaryngology and pathology physicians for routine histopathologic examination after tonsillectomy would contribute to an early diagnosis of rare tonsillar malignancies in patients with a real asymmetry.
